# Effectiveness of Extracellular Vesicle Application in Skin Aging Treatment and Regeneration: Do We Have Enough Evidence from Clinical Trials?

**DOI:** 10.3390/ijms26052354

**Published:** 2025-03-06

**Authors:** Anna Domaszewska-Szostek, Marta Krzyżanowska, Agnieszka Polak, Monika Puzianowska-Kuźnicka

**Affiliations:** 1 Department of Human Epigenetics, Mossakowski Medical Research Institute, Polish Academy of Sciences, 02-106 Warsaw, Poland; 2 Department of Ophthalmology, Collegium Medicum, Nicolaus Copernicus University, 85-094 Bydgoszcz, Poland; 3 School of Clinical Medicine, University of Cambridge, Addenbrooke’s Hospital, Hills Rd, Cambridge CB2 0SP, UK; 4 Department of Geriatrics and Gerontology, Medical Centre of Postgraduate Education, 01-826 Warsaw, Poland

**Keywords:** extracellular vesicles, exosomes, skin aging, clinical trials, regenerative medicine

## Abstract

In recent years, there has been a dynamic development in therapies utilizing extracellular vesicles (EVs) including exosomes. Therefore, we have conducted an analysis of the scientific literature to verify the current state of knowledge about these therapies. A total of 12 clinical studies were analyzed, covering the use of EVs in treating skin aging, acne scars, alopecia, and wound healing. The results indicate that EVs and exosomes hold potential in regenerative skin therapies, offering innovative and non-invasive therapeutic approaches. At the same time, significant challenges related to the standardization of their production and the lack of large-scale randomized studies were identified. Thus, we also evaluated the investigated clinical trials in regard to the MISEV (Minimal Information for Studies of Extracellular Vesicles) criteria. This review provides a comprehensive overview of the contemporary applications of EVs in skin therapy and regenerative medicine, highlighting directions for further research.

## 1. Introduction

The skin serves as a highly organized border between the body and the external environment. However, beyond functioning as a mechanical barrier that protects against environmental factors, it actively participates in numerous processes involving the function of neuroendocrine and immune systems [1,2,3]. This complex network enables the skin not only to perceive stimuli, such as ultraviolet radiation (UVR), microbial pathogens, environmental pollutants, or mechanical injuries, but also to respond appropriately through a system of neuropeptides, neurotransmitters, and locally secreted steroid hormones [2]. As a result, the skin possesses adaptive mechanisms that facilitate its response to various forms of environmental stress, playing a crucial role in maintaining the integrity and homeostasis of the entire organism [4].

One of the key components of this adaptive capacity is the complex neuroimmunoendocrine network, which comprises cutaneous nerve fibers, immune cells (including mast cells and Langerhans cells), and specialized cell populations within the epidermis (keratinocytes) and dermis (fibroblasts) [5,6,7]. Together, these elements regulate essential processes such as cutaneous inflammation, wound healing, melanogenesis, and water-lipid homeostasis, which influence skin hydration and elasticity. During aging, the role of this system becomes particularly evident [8]. Neurohormonal and immune alterations, exacerbated by chronic stress, UVR, and environmental pollutants, can disrupt the balance between inflammatory responses and tissue regeneration [9]. This imbalance, in turn, accelerates the aging process, leading to loss of elasticity, impaired barrier function, and wrinkle formation [6,9,10].

A key example of the close integration between skin function and neuroendocrine mechanisms is its local equivalent of the hypothalamic–pituitary–adrenal (HPA)-like axis [6]. Under physiological conditions, the skin produces corticotropin-releasing hormone (CRH), proopiomelanocortin (POMC)-derived peptides, such as adrenocorticotropic hormone (ACTH) and α-melanocyte-stimulating hormone (α-MSH), as well as glucocorticoids within a tightly regulated local circuit [11]. The skin can specifically modulate immune responses by altering cytokine profiles. Additionally, it regulates both pro- and anti-inflammatory reactions, as well as melanogenesis in melanocytes, by influencing pigmentation and providing protection against UVR [12]. However, when factors such as UVR, psychological stress, or chemical toxins act chronically and excessively, the initially beneficial and adaptive skin response becomes disrupted [10,12]. This leads to a breakdown of regulatory feedback mechanisms, while sustained overproduction of inflammatory mediators and reactive oxygen species (ROS) can trigger a cascade of damage to collagen fibers and other components of the extracellular matrix. As a result, features of inflammaging, a chronic, low-grade inflammatory state associated with aging, become evident, along with dermal remodeling and the progression of changes leading to wrinkle formation, loss of firmness, and hyperpigmentation [13]. Furthermore, an overactivated neuroimmunoendocrine system may exacerbate pre-existing inflammatory skin disorders, such as psoriasis or atopic dermatitis, contributing to a more severe and treatment-resistant disease course [14].

UVR is a key environmental factor influencing skin physiology, with both beneficial and detrimental effects. On one hand, controlled UVR exposure is essential for vitamin D synthesis, which plays a crucial role in calcium homeostasis, immune regulation, and overall skin health, among others [15]. Additionally, UVR can induce mild hormetic stress responses that activate repair mechanisms and enhance antioxidant defenses, potentially contributing to skin resilience [16]. However, chronic or excessive UVR exposure remains a primary driver of skin photoaging, inflammation, and carcinogenesis [4,12].

At the molecular level, UVR induces oxidative stress through the generation of ROS, leading to DNA damage, lipid peroxidation, and mitochondrial dysfunction in skin cells [17]. This oxidative burden accelerates collagen degradation by upregulating matrix metalloproteinases (MMPs), particularly MMP-1 and MMP-3, while downregulating type I and III collagen synthesis [3,18]. These molecular alterations contribute to the formation of fine lines, deep wrinkles, skin dehydration, and pigmentation disorders. Furthermore, UVR disrupts the skin microbiome, leading to an imbalance in commensal bacteria and promoting inflammatory responses that exacerbate tissue damage [19].

On a cellular level, chronic ultraviolet (UV) exposure contributes to cellular senescence through the activation of the p53 and p16^INK4a^ pathways, leading to growth arrest and the secretion of pro-inflammatory cytokines such as IL-6 and TNF-α [20]. This phenomenon, termed the senescence-associated secretory phenotype (SASP), amplifies local inflammation, further impairing extracellular matrix integrity and reducing the regenerative capacity of dermal fibroblasts [21]. UVB radiation, in particular, induces direct DNA damage by forming cyclobutane pyrimidine dimers (CPDs), which, if not efficiently repaired, increase the risk of photoaging and skin malignancies [20].

In response to these challenges, modern dermatology and aesthetic medicine are actively seeking innovative strategies to support skin regeneration and delay the aging process [10]. There is growing interest in therapies based on biological mechanisms of intercellular communication, which can modulate the inflammatory response, stimulate collagen synthesis, and promote skin repair. In this context, extracellular vesicles (EVs) playing a crucial role in cell-to-cell signaling deserve special attention [22,23].

EVs, and more specifically exosomes, have generated significant interest in aesthetic medicine, with numerous exosome-based therapies already in use. However, despite the increasing interest in these treatments, the available clinical evidence on their effectiveness remains limited. This has led us to undertake a comprehensive review of the current scientific understanding regarding the role of EVs in aesthetic and dermatological applications.

EVs are lipid-bilayer-enclosed particles released from all living cells [24]. These vesicles are characterized by their inability to replicate independently, as they do not contain a functional nucleus. To be classified as an EV, a particle must meet the following criteria: it must be derived from a cell, enclosed by a lipid membrane, and incapable of self-replication [25].

Exosomes are specific types of EVs, typically smaller in size: 30–150 nm [26]. The formation of exosomes involves the budding of vesicles within multivesicular bodies (MVBs), which subsequently fuse with the plasma membrane, releasing the vesicles into the extracellular space. The term “exosome” specifically refers to those EVs that arise from the endosomal network, distinguishing them from other types of EVs arising from different cellular processes [25,27].

EVs are emerging as one of the most promising therapeutic tools in contemporary regenerative medicine. Both exosomes [28] and EVs more generally [29] play a critical role in intercellular communication. Their lipid bilayer structure enables the efficient transport of bioactive molecules—proteins, RNA, microRNA, lipids, and DNA—to target cells over long distances without degradation, even in enzyme-rich environments such as blood and urine [30,31]. This capacity to deliver bioactive compounds through enzyme-rich fluids makes them particularly suited for therapeutic applications, allowing them to reach tissues that are otherwise difficult to access [32,33].

In regenerative medicine, exosomes have shown efficacy in skin rejuvenation, wound healing, hyperpigmentation treatment, and cartilage repair [34]. Moreover, in dermatology, the vesicles derived from stem cells or fibroblasts enhance collagen production, improve skin elasticity, and reduce inflammation, promoting visibly healthier and more youthful skin. Furthermore, exosomes derived from mesenchymal stem cells (MSCs) show promise in treating alopecia by stimulating hair follicle growth and extending the anagen phase, offering a minimally invasive alternative to hair transplantation [35]. Their ability to bypass immune responses and cross biological barriers, such as the blood-brain barrier, further establishes exosomes as versatile vehicles for delivering growth factors, cytokines, and gene-editing tools like clustered regularly interspaced short palindromic repeats (CRISPR) [36].

EVs, conserved across species, are critical for intercellular communication and hold promise for advanced therapies like drug delivery and gene therapy [37]. Moreover, exosomes support tissue repair and regeneration by modulating key processes, including angiogenesis, inflammation, skin regeneration, and collagen synthesis. They facilitate the targeted delivery of signaling molecules such as transforming growth factor beta (TGF-β), Wnt/β-catenin, vascular endothelial growth factor (VEGF), fibroblast growth factor (FGF), epidermal growth factor (EGF), and platelet-derived growth factor (PDGF), which regulate fibroblast activation, collagen production, and epithelialization [33,38].

Also, exosomal miRNAs have demonstrated significant roles in various regenerative processes, including wound healing, hair restoration, and skin aging. For example, miR-1246, found in exosomes, has been shown to activate the TGF-β/Smad signaling pathway, which enhances collagen production and helps prevent the apoptosis of skin cells. Moreover, miR-767 has been identified as an important player in delaying the aging process by regulating fibroblast function and supporting skin regeneration [39]. miR-125b, also found in exosomes, has been shown to enhance angiogenesis, a crucial process for wound healing. Additionally, miR-146a, another exosomal miRNA, has been found to reduce inflammatory cytokines and promote neovascularization, facilitating faster tissue regeneration and wound closure. These miRNAs, delivered via exosomes, help modulate the inflammatory response and support tissue repair mechanisms, making them promising candidates for improving wound healing [40]. In the context of alopecia, miR-122-5p, carried by exosomes, has been identified for its ability to stimulate hair follicle stem cells and regulate the hair growth cycle. This miRNA helps reactivate dormant follicles, promoting hair regrowth. miR-122-5p can target key genes involved in hair follicle regeneration, making it a potential therapeutic option for treating hair loss [41].

EVs and exosomes originate from diverse biological sources, including MSCs, epithelial cells, adipocytes, and platelets, offering unique therapeutic advantages. For example, MSC-derived exosomes exhibit potent anti-inflammatory and regenerative properties, making them ideal for skin rejuvenation and wound healing [31,42]. Their cell-free nature eliminates risks associated with cellular therapies, such as immunogenicity and transplant-related complications [42]. Exosome-based therapies stimulate natural biological processes, offering a holistic approach to skin regeneration [43].

**Figure 1:**
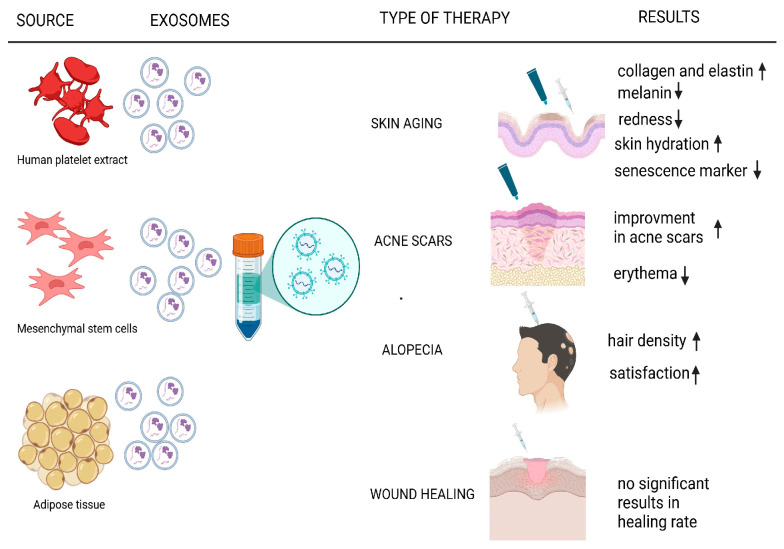
Current applications of EVs in regenerative and aesthetic medicine. ↑ means increase, ↓ means decrease.

Despite their transformative potential, EV-based therapies face significant challenges, including the lack of standardized methods of production and isolation, storage, and administration protocols, high production costs, and the limited number of large-scale clinical trials validating their long-term safety and efficacy [44,45]. This review aims to critically evaluate the current applications of EVs in regenerative and aesthetic medicine, focusing on their roles in anti-aging therapies, acne scar regeneration, alopecia treatment, and wound healing (Figure 1), by synthesizing published clinical trials and research identifying the benefits, limitations, and future directions for exosome-based therapies in this rapidly advancing field.

## 2. Results

### 2.1. Anti-Aging Therapies

Skin aging is a multifactorial process driven by intrinsic genetic and epigenetic mechanisms, as well as extrinsic environmental stressors including UVR, pollution, and oxidative stress [46,47]. A hallmark of this process is the accumulation of senescent cells, which secrete pro-inflammatory cytokines and matrix metalloproteinases, accelerating collagen and elastin degradation [48]. These changes result in reduced skin elasticity, wrinkle formation, and impaired regenerative capacity. Targeting these mechanisms through senolytic therapies, epigenetic modulation, or antioxidant strategies holds potential for mitigating skin aging and preserving homeostasis [49].

Epigenetic modifications, such as DNA methylation and histone alterations, significantly influence skin aging by dysregulating gene expression and impairing cellular function [50,51]. Age-associated epigenetic drift has been linked to reduced collagen synthesis and structural degradation of the dermal matrix [52]. Cellular senescence further exacerbates aging by fostering chronic inflammation through the senescence-associated secretory phenotype (SASP), which disrupts tissue integrity and accelerates dermal deterioration [21].

Extrinsic aging, primarily shaped by environmental exposures, is increasingly recognized as a critical factor in skin aging. Chronic UV exposure generates ROS, leading to DNA damage and activation of matrix-degrading pathways. Pollutants, including particulate matter and toxins, penetrate the skin barrier, triggering oxidative stress and inflammatory responses that further accelerate aging [53,54].

Several recent reviews provide comprehensive insights into the molecular mechanisms underlying skin aging and potential therapeutic approaches. López-Otín et al. outline the key hallmarks of aging, including genomic instability, telomere attrition, epigenetic alterations, and loss of proteostasis, while discussing emerging relevant treatment strategies [55]. Krutmann et al. focus on dermal aging, highlighting fibroblast dysfunction and extracellular matrix degradation, and evaluating existing and novel anti-aging interventions [56]. Zhang et al. emphasize the role of natural compounds in counteracting oxidative stress and inflammation, underscoring their therapeutic potential [57]. Collectively, these analyses offer a valuable foundation for advancing skin rejuvenation research and developing targeted anti-aging therapies.

Exosomes can influence critical cellular processes involved in skin rejuvenation, making them a promising tool in anti-aging therapies. These nano-sized vesicles carry bioactive molecules, which can regulate the synthesis of collagen, elastin production, and cell proliferation. Modulation of pathways such as TGF-β signaling allows exosomes to promote the repair and regeneration of aged and damaged skin, improving elasticity, hydration, and overall skin texture, as well as reducing wrinkles. Moreover, they can reduce oxidative stress and inflammation, further mitigating visible signs of aging [58].

Cho et al. [59] investigated the effects of exosomes derived from human adipose tissue stem/stromal cells (ASC) on skin hyperpigmentation in a split-face study involving 21 female participants aged 39 to 55 years (mean age and information about Fitzpatrick skin type not available). The participants applied ASC exosomes topically over an 8-week period, with one side of the face treated with the exosome formulation and the other with a placebo. The study demonstrated a statistically significant reduction in melanin content on the treated side compared to the placebo. The most pronounced effects were observed during the initial weeks of therapy, highlighting the potential of ASC exosomes in addressing skin hyperpigmentation and improving skin brightness. However, the researchers noted a plateau in efficacy over time, which they attributed to potential limitations in transdermal delivery mechanisms. To address this, Cho et al. emphasized the importance of further research to optimize ASC exosome formulations. Enhancements in delivery systems, such as nanoparticle encapsulation or microneedling, were suggested as potential strategies to improve penetration and sustain the therapeutic effects. These findings underscore the promising role of ASC exosomes in cosmetic dermatology, particularly in treatments targeting uneven skin tone and pigmentation disorders.

Research into the regenerative potential of platelet-derived exosomes has expanded with the work of Proffer et al. [60], who provided evidence for the efficacy of platelet-derived exosomes in aesthetic treatments through a prospective, single-arm, non-randomized longitudinal study. This investigation enrolled 56 participants (48 females and 8 males) aged 40 to 80 years (mean age: 54 ± 11), assessing the safety and efficacy of a novel serum enriched with human platelet extract (HPE), branded as the Intensive Repair Serum from Rion Aesthetics, LCC (Rochester, MN, USA). The participants in this study were classified as Fitzpatrick skin types I to IV; however, the exact distribution of participants across these skin types was not specified. Participants adhered to a standardized skincare regimen, applying the serum twice daily for six weeks, alongside complementary skincare products. The study employed VISIA-CR Generation 5 imaging for quantitative evaluation, which revealed statistically significant improvements in overall skin health. Key outcomes included a reduction in erythema, wrinkles, and melanin production, coupled with enhancements in luminosity and color evenness. The mean improvement in skin health score was 224.2 ± 112.8 (*p* ≤ 0.0001), correlating with significant progress across various metrics of facial photodamage. Further analysis focused on the top quartile of responders, who demonstrated maximal improvement. These participants exhibited a 440% increase in skin health parameters, including a 5.42 ± 1.36 unit improvement in luminosity (*p* ≤ 0.0001) and enhanced color evenness. Static wrinkle reduction was observed across multiple facial regions, with significant improvements in fractional areas of the forehead, periorbital, and perioral wrinkles. Patient-reported outcomes reinforced the quantitative data, with 98.2% of participants expressing a willingness to continue treatment. The serum was well-tolerated, with only mild dryness reported in 16.1% of participants, likely exacerbated by winter conditions during the study period. No severe adverse events were recorded. Proffer et al. [60] concluded that platelet-derived exosome products represent a transformative advancement in aesthetic medicine, offering a consistent, safe, and effective approach to skin rejuvenation.

Wyles et al. [44] conducted further studies on the HPE product, evaluating its efficacy in treating signs of skin aging. Their 2024 study compared HPE, an allogenic product derived from leukocyte-depleted platelets, with vitamin C serum in anti-aging therapy. The study involved 60 participants aged 40 to 80 years (mean age: 52.5), the majority of whom had Fitzpatrick skin type II (83.3%), with smaller proportions of types III, IV, and V, and no type I or VI. The participants applied HPE to the dorsum of their right hand and vitamin C serum to their left, twice daily for 12 to 26 weeks. Both therapies demonstrated comparable effectiveness in reducing signs of aging, including improved skin texture and tone. However, HPE showed greater tolerability, particularly among individuals with sensitive skin, suggesting it is a viable alternative to vitamin C, the long-considered gold standard in anti-aging treatments.

Building on these findings, Wyles et al. [41] conducted a study involving 56 participants aged 40 to 80 years old (mean age: 54) to evaluate the efficacy of a 12-week facial topical application of HPE. Participants were selected across all Fitzpatrick skin types, with nine individuals classified as type III or higher. However, no detailed distribution by skin type was provided. Participants applied a standardized dose of 1 mL of HPE to their facial skin twice daily. Regenerative exosome-based approaches, such as HPE, showed substantial benefits in improving skin roughness, hydration, and reduction of wrinkle depth, particularly in sensitive skin types. This was supported by the participants’ subjective assessments and objective scoring metrics, further underscoring HPE’s potential in anti-aging therapy. Furthermore, standard 5 mm punch biopsies were obtained from the inner aspect of the upper arm of 20 participants before treatment and after 12 weeks of the study. The method of selecting these participants from the total cohort of 56 was not specified. The histopathological results demonstrated an increase in collagen fiber thickness and elastin fiber formation in the dermis after 12 weeks of HPE use, indicating an improvement in skin structure. These findings were based on a direct comparison of histopathological results between baseline and post-treatment samples.

Both studies highlighted the regenerative properties of HPE, emphasizing its ability to enhance skin health through mechanisms that promote cellular repair and extracellular matrix restoration. These findings position HPE as a promising candidate for incorporation into aesthetic and dermatological practices, offering a safe and effective approach to skin anti-aging therapy.

Another Rion Aesthetics product utilized for evaluating skin recovery after laser resurfacing is the Calm Serum. A study conducted by Dayan et al. [61] aimed to determine whether the HPE serum could reduce downtime and improve clinical outcomes during the post-fractional CO_2_ laser recovery phase, in a 1-month study period. The study included 18 female participants aged 32–77 years (mean age: 55.2), divided equally into the experimental and control groups. However, the study design lacked the robustness of a split-face comparison, and the exclusion of darker skin types (Fitzpatrick IV–VI) and male participants limits the generalizability of the findings. The results demonstrated that the serum had a statistically significant positive impact on skin recovery. Participants in the HPE-treated group experienced reduced crusting by day 10, with an average discomfort rating of 0.11 compared to 0.89 in the control group (*p* = 0.0193). Downtime was significantly shorter in the HPE group, with fewer symptoms of redness, tenderness, and irritation reported over the first 14 days (*p* = 0.03). Additionally, by day 14, participants treated with the serum reported brighter and more youthful-looking skin compared to the control group (*p* = 0.007 and *p* = 0.003, respectively).

Svolacchia et al. [45] investigated the efficacy of exosomes and signaling nanovesicles derived from autologous adipose tissue in skin regeneration and anti-aging treatments. This clinical trial included 72 female participants aged 34 to 68 years (mean age: 48) and focused on the application of exosome-enriched formulations. However, the article does not specify the distribution of Fitzpatrick skin types among the participants. The exosomes were isolated using Skin-B^®^, a sterile solution containing amino acids and non-viscoelastic macromolecular hyaluronic acid. This protocol aimed to optimize tissue regeneration by leveraging signaling vesicles containing mRNA, microRNA, growth factors, and bioactive peptides. The final product, containing nanovesicles with an average size of 90 nanometers, was injected into the dermis using a mesotherapy technique. Clinical outcomes were evaluated using the Berardesca Scale, the Numeric Rating Scale (NRS), and the Modified Vancouver Scale (MVS). These assessments demonstrated significant improvements in skin parameters, including wrinkle reduction, enhanced softness, hydration, and stability. At follow-up intervals of 15, 30, and 90 days, participants reported high satisfaction, supported by statistically significant improvements in all measured outcomes (*p* < 0.0001). This treatment showed some improvement in the signs of photoaging and tissue aging without apparent adverse effects. The study concluded that exosome-enriched therapies may provide a minimally invasive approach to skin rejuvenation. However, further research is needed to fully assess the safety, efficacy, and long-term outcomes of this technique before it can be considered a reliable option in aesthetic medicine and regenerative dermatology.

The synergistic potential of combining exosome-based therapies with other treatments was demonstrated in a 12-week prospective, randomized, split-face study conducted by Park and Kwon, et al. [62]. This investigation evaluated the efficacy of microneedling in combination with a human adipose stem cell-derived exosome-containing solution (HACS) for the treatment of facial skin aging. To prepare the HACS, the authors mixed 2 mL normal saline solution with a vial of ASCE+ Derma Signal Skin Rejuvenation Lyophilized Vial (SRLV)-S (ExoCoBio Inc., Seoul, Republic of Korea).

The study enrolled 28 participants, including 20 women and 8 men, aged between 43 and 66 years (mean age: 54). Twelve (42.9%) had Fitzpatrick skin type III, while sixteen (57.1%) had type IV. All participants completed the study, which consisted of three treatment sessions performed at three-week intervals and a six-week follow-up period after the final intervention. In the experimental protocol, the HACS was applied to one side of the face, followed by microneedling to enhance dermal penetration of the solution. The opposite side of the face, serving as a control, was treated with normal saline and microneedling. Clinical outcomes were assessed using the Global Aesthetic Improvement Scale (GAIS), with significant differences favoring the HACS-treated side observed at weeks 6 and 12. By the final follow-up, the HACS-treated side exhibited a statistically significant improvement in overall skin appearance compared to the control side (*p* = 0.005). Objective measurements further confirmed the superior efficacy of the HACS treatment in improving wrinkles, elasticity, hydration, and pigmentation. Wrinkle parameters, including average roughness (Ra), showed a 12.4% reduction on the HACS-treated side compared to a 6.6% reduction on the control side. Similarly, skin elasticity increased by 11.3% from baseline on the HACS-treated side, while the control side experienced a decline of 3.3%, with this difference reaching statistical significance (*p* = 0.002). Skin hydration also improved, with a 6.5% increase observed on the HACS-treated side compared to 4.5% on the control (*p* = 0.037). In terms of pigmentation, the melanin index decreased by 9.9% on the HACS-treated side versus 1.0% on the control side, further highlighting the effectiveness of the exosome-based treatment (*p* = 0.044). Histopathological analyses of skin biopsies supported these findings, revealing an increased density of collagen and elastic fibers, as well as greater mucin deposition in the HACS-treated skin compared to baseline and the control side. Importantly, no serious adverse events were reported during the study. Transient erythema and petechiae, observed in some participants, resolved spontaneously within one week. Park et al. concluded that the combination of a HACS and microneedling provides significant synergistic benefits in facial skin rejuvenation. This combined approach enhances multiple aspects of skin health, including wrinkle reduction, improved elasticity, hydration, and pigmentation.

A study conducted by Ye et al. [63] provided significant evidence supporting the efficacy of human mesenchymal stem cell (hMSC)-derived exosomes in improving skin sensitivity and uniformity. This prospective, single-arm, open-label study enrolled 22 female participants aged 24 to 55 years (mean age: 40), of whom 20 completed the trial. Participants, diagnosed with sensitive skin and exhibiting symptoms such as dryness, itching, burning, or tingling, were required to have a lactic acid stinging test score of 3 or higher.

They applied an hMSC-derived exosome formulation topically to the facial skin twice daily for 28 days. The clinical outcomes demonstrated notable improvements in objective and subjective skin health measures. By day 28, scores for skin roughness, erythema, dryness, and sensations of tension or itching decreased significantly compared to baseline (*p* < 0.05). Instrumental assessments revealed reductions in transepidermal water loss (TEWL), erythema (a* value), sebaceous secretion, and improved hydration and pH balance, indicating restored skin barrier functionality. Histological analysis further highlighted the regenerative effects of the exosome formulation, including enhanced fibroblast proliferation and migration, coupled with increased collagen deposition. Participants also reported high levels of satisfaction with the treatment, with 80% rating the product as very satisfactory at the end of the study. Importantly, no adverse events were recorded, confirming the safety and tolerability of the formulation. The authors concluded that hMSC-derived exosomes represent a promising, non-invasive alternative for managing sensitive skin and restoring barrier function. These findings indicate that exosomes could be a viable alternative to more aggressive treatments, particularly for patients seeking less invasive options. Additionally, incorporating molecular analyses such as their impact on fibroblasts and angiogenesis could provide deeper insights into the mechanisms driving their therapeutic effects. Further studies are warranted to explore their molecular mechanisms, including their impact on fibroblasts and angiogenesis, and to compare their efficacy with other standard treatments.

### 2.2. Acne Scar Regeneration

Acne scars present a significant therapeutic challenge because of their permanence and the difficulty in skin regeneration. They form due to inflammatory acne lesions causing damage to the dermal extracellular matrix. This damage disrupts the balance of collagen synthesis and degradation, leading to structural irregularities in the skin with either excessive deposition or insufficient repair. Research indicates that exosome-based therapies offer promising solutions for managing acne scars. Those nano-sized particles activate fibroblasts by growth factors such as TGF-β, leading to enhanced collagen and elastin production, thereby improving ECM integrity and skin structure. Moreover, they can contribute to the reduction of chronic inflammation, by delivering anti-inflammatory cytokines, such as TGF-β, IL-10, and downregulating pro-inflammatory pathways, which hinders scar healing. Also, exosomes deliver molecules such as VEGF, which contributes to angiogenesis and thus blood flow to the damaged tissues. Lastly, their ability to regulate cell proliferation contributes to the wound-healing process [38].

The synergistic action of exosomes combined with laser technologies appears particularly promising. The results of Kwon et al. [43] highlight the enhanced regenerative potential of combining adipose tissue stem cell-derived exosomes (ASCE) with fractional CO_2_ lasers for the treatment of atrophic acne scars. This study was conducted as a 12-week, prospective, double-blind, randomized, split-face trial involving 25 Korean participants (18 males and 7 females) aged 19 to 54 years (mean age: 35.6 years), with Fitzpatrick skin types III and IV. All participants received three sessions of fractional CO_2_ laser treatment, with post-treatment application of either ASCE gel or a placebo gel on randomized halves of the face. Clinical outcomes were assessed using the Échelle d’Évaluation Clinique des Cicatrices d’Acné (ECCA) scoring system and the Investigator’s Global Assessment (IGA) scale. At the final follow-up, the ASCE-treated side showed a significantly greater reduction in ECCA scores compared to the control side (32.5% vs. 19.9%, *p* < 0.01). Participants reported a milder post-treatment erythema and shorter downtime on the ASCE-treated side compared to the control side (4.1 vs. 4.3 days, *p* = 0.03), emphasizing the enhanced safety profile of the combined therapy. Additionally, ASCE-treated areas exhibited superior improvement in IGA grades, with 16 out of 25 participants achieving a grade of 2 or higher, compared to only 12 on the control-treated side. The study concluded that the synergistic effects of ASCE and fractional CO_2_ laser therapy significantly enhance the efficacy and safety of atrophic acne scar treatments. However, the authors noted the need for further studies to optimize the dosing regimen and application frequency of ASCE to maximize clinical outcomes.

### 2.3. Alopecia Treatment

Alopecia, particularly the androgenetic type, involves hair thinning due to follicle miniaturization. The hair growth phase shortens due to factors such as inflammation, oxidative stress, vascularization, and dihydrotestosterone action, whilst the resting phase prolongs, resulting in a reduced follicle size and output. Exosomes are emerging as a promising treatment by modifying the key pathological factors. With their immunomodulatory and anti-inflammatory effects, they aid hair regeneration. It has been shown that in mice, exosomes slow the progression of alopecia through decreasing T helper cell proliferation and increasing immunoregulatory mRNAs, including forkhead box protein P3 (FoxP3) and arginase 1 [64]. Moreover, they deliver growth factors such as VEGF, insulin-like growth factor 1 (IGF-1), and FGF, stimulating dermal papilla cells and extending the anagen phase of hair growth. They also contribute to angiogenesis around hair follicles and mitigate oxidative stress, protecting follicular cells. Lastly, they activate the Wnt/β-catenin pathway, contributing to follicle development and cycling [65].

Ersan et al. [35] investigated the efficacy of MSC-derived exosomes in the treatment of androgenic alopecia through a prospective study involving 30 male participants aged 22 to 65 years (mean age: 34.65). Participants were classified as hair type III to VI on the Norwood–Hamilton scale. The study used exosomes derived from neonatal foreskin MSCs, isolated under good manufacturing practice (GMP) conditions, with a size distribution of 139.7 ± 2.3 nm as confirmed by nanoparticle tracking analysis (NTA). The treatment protocol involved multiple microinjections of a total of 3 mL of exosomes, with 2 mL administered to the frontal area and 1 mL to the vertex region (1010 extracellular vesicles per 1 mL), using the nappage technique, a form of mesotherapy. Hair density was assessed pre-treatment and at the 4th and 12th weeks post-treatment using digital dermatoscopy and TrichoScan analysis. Significant increases in hair density were observed, with mean values rising from 149.7 ± 13.7 hairs/cm² at baseline to 153.6 ± 16.8 hairs/cm² at 4 weeks (*p* = 0.043) and further to 157 ± 18.3 hairs/cm² at 12 weeks (*p* = 0.002). Histological analyses showed increased hair follicle density and signs of follicular regeneration. Patient satisfaction surveys, conducted at 4 and 12 weeks, demonstrated a statistically significant improvement in perceptions of reduced hair loss and new hair growth at the later follow-up (*p* < 0.05). Participants reported no adverse effects throughout the study, highlighting the safety and tolerability of this therapy. Ersan et al. concluded that foreskin-derived MSC exosome injections offer a promising, minimally invasive approach to treating androgenic alopecia, with sustained improvements in hair density and patient satisfaction over time. However, the authors emphasized the need for future research to explore optimal dosing strategies and the effects of repeated exosome treatments in larger, randomized controlled trials.

Compared to conventional therapies such as minoxidil or finasteride, MSC-derived exosomes have demonstrated competitive efficacy, with significant improvements in hair density observed as early as four weeks post-treatment. However, despite these encouraging results, the absence of long-term studies limits understanding of the durability of these effects and potential side effects. Standardizing exosome administration methods, including determining optimal doses and therapeutic session frequencies, remains critical for future research. Additionally, further investigations into the bioavailability of exosomes following injections could provide deeper insights into their mechanisms of action and overall efficacy.

### 2.4. Wound Healing

Wound healing is a multifaceted process involving inflammation, tissue remodeling, and regeneration, all of which EV can enhance. They contribute to reducing inflammation and promoting angiogenesis through various mechanisms. Additionally, they deliver growth factors such as epidermal growth factor (EGF) and FGF, which stimulate fibroblast proliferation, collagen synthesis, and keratinocyte migration, key processes for effective tissue repair [66].

Johnson et al. [42] conducted the first human clinical trial to assess the safety and potential efficacy of allogeneic platelet-derived extracellular vesicles (termed pEVs) in the context of delayed wound healing. The study was a phase I, double-blind, placebo-controlled trial involving 11 healthy adult participants (mean age: 29 years). Each participant received two 4 mm punch biopsies on the inner upper arms leaving wounds to the skin, with one wound randomly assigned to receive a subcutaneous injection of pEVs (100 µg in 340 µL) and the other treated with a placebo. The pEVs were manufactured using a novel ligand-based exosome affinity purification (LEAP) process, which ensures clinical-grade product consistency and bioactivity. The study’s primary objective was to evaluate the safety of pEVs in humans. No severe adverse events or abnormalities were reported, confirming the favorable safety profile of pEVs. Both the pEV-treated and placebo-treated wounds exhibited complete healing within 30 days, with a mean healing time of 22.8 ± 8.7 days in both groups. The lack of observed differences in wound closure rates was anticipated, given the healthy status of the participants and the naturally rapid healing of small, acute wounds. Secondary exploratory objectives included the assessment of pEV bioactivity in wound healing. Although no significant differences were observed in healing rates between the groups, the pEV-treated wounds showed evidence of cellular engagement. In vitro assays conducted in parallel with the clinical trial demonstrated that pEVs promoted fibroblast proliferation, migration, and angiogenesis by activating the ERK and Akt signaling pathways—key mechanisms involved in tissue regeneration. The findings from this trial underscore the safety of LEAP-manufactured pEVs and their potential as a therapeutic for delayed wound healing. However, the authors emphasized the need for future studies targeting patient populations with chronic wounds or impaired healing conditions, where the regenerative properties of pEVs may have a more pronounced therapeutic effect.

Preclinical models using MSC-derived exosomes have further highlighted their therapeutic potential, demonstrating accelerated epithelialization and reduced inflammatory responses in damaged tissues. These findings align with earlier studies emphasizing exosomes’ roles in angiogenesis and fibroblast proliferation, both critical for successful wound healing. Johnson et al. [42] also noted that platelet-derived exosomes reduce inflammatory responses, supporting their potential use for treating chronic wounds, such as diabetic ulcers.

Despite these promising results, clinical trials involving small patient cohorts limit the generalizability of the findings. Future research should focus on elucidating molecular mechanisms, such as modulating pro-inflammatory cytokine levels, to understand exosomes’ therapeutic effects better. Additionally, investigating their impact on bacterial biofilms, a common complication in chronic wounds, represents a crucial avenue for further study. Such efforts could enhance the clinical application of exosomes in managing complex cases, including diabetic wounds and pressure ulcers.

### 2.5. Current Research Trends

ClinicalTrials.gov lists 24 current studies focusing on skin conditions where the treatment of choice is exosomes and one study specifically on skin rejuvenation, highlighting the growing interest in exosome-based therapies for dermatological applications. These studies explore a range of conditions, including atopic dermatitis, psoriasis, chronic wounds, and skin aging, emphasizing the versatility of exosomes in addressing diverse skin-related challenges. The trials predominantly utilize exosomes derived from MSCs and platelet-rich plasma, valued for their regenerative and reparative properties. Delivery methods include topical applications, favored for their non-invasive nature, and subcutaneous injections, which target deeper tissue layers [67,68]. Most trials are in early phases, assessing safety, tolerability, and preliminary efficacy to establish a foundation for larger-scale studies. A notable trend involves combining exosome therapies with adjunctive treatments such as microneedling or laser therapy to enhance outcomes. In the context of skin rejuvenation, exosomes are being evaluated for their ability to stimulate collagen synthesis and improve skin elasticity, offering a promising anti-aging strategy. Collectively, these trends underscore the potential of exosomes to provide innovative and effective solutions for both chronic and refractory skin conditions.

Also, plant-derived exosomes are increasingly being incorporated into cosmetic and aesthetic medicine formulations, often promoted for their anti-aging, regenerative, and moisturizing properties. However, as highlighted by Cho et al. [69], significant differences exist between the mechanisms of action of plant exosomes and traditional plant extracts, emphasizing the need for more robust research to evaluate their safety and efficacy. The authors demonstrated that exosomes have a significantly stronger impact on keratinocyte transcriptome than plant extracts, suggesting their superior potential as active ingredients in cosmeceuticals. Specifically, in the analysis of genes associated with aging, regeneration, skin barrier function, and hydration (e.g., MMP12, MMP13, NOTCH3, FGF12), the exosome-treated group was predicted to promote skin health more effectively than the extract-treated group. Despite these promising findings, many commercial products containing plant-derived exosomes lack scientific evidence to support their claimed benefits. Clinical validation of their efficacy for skin health remains in its early stages. Therefore, well-designed, comprehensive studies are essential to substantiate the proposed advantages of plant-derived exosomes in aesthetic medicine and to establish their therapeutic potential fully.

## 3. Discussion

Extracellular vesicles have shown great promise in regenerative medicine, particularly in dermatology and aesthetic treatments. However, despite promising preclinical and early clinical data, their successful translation into widespread clinical use is hindered by several key challenges. The main obstacles include limitations in large-scale production, batch-to-batch variability, regulatory hurdles, and the need for standardized therapeutic formulations. Unlike synthetic drug carriers, EVs are biologically derived, leading to inconsistencies in their composition, therapeutic efficacy, and stability. These challenges must be addressed to facilitate their safe and effective application in clinical settings. [70,71]

While exosomes’ therapeutic potential is promising, ensuring their safe and effective application requires addressing several methodological challenges and standardizing research protocols. This is crucial to accurately assessing their efficacy and safety in clinical settings. Therefore, we decided to evaluate whether the data presented in our review meet the criteria outlined in the Minimal Information for Studies of Extracellular Vesicles (MISEV), a set of comprehensive guidelines (ISEV, 2023) [72]. The results of this analysis are presented in Table 1.

The studies regarding the HPE products of Rion Aesthetics (Intense Repair Serum and Calm Serum), as well as the ASCE of ExoCoBio Inc., were not included in the comparison. While the products claim to contain exosomes, the studies did not describe the isolation methods, molecular markers, or purity assessments of these EVs. This omission undermines the reliability of attributing observed effects directly to exosomal activity. The funding and involvement of the product’s manufacturer (Rion Aesthetics, Rochester, MN, USA; ExoCoBio Inc., Seoul, South Korea) raise concerns about potential conflicts of interest, particularly given the studies’ reliance on proprietary technology.

MISEV is a set of comprehensive guidelines established by the International Society for Extracellular Vesicles (ISEV) to promote methodological rigor, transparency, and reproducibility in EV research. Currently, in its 2023 iteration (MISEV2023), the guidelines outline the minimum requirements for nomenclature, sources, isolation methods, characterization, and functional studies of EVs. Additionally, they advocate for multi-step approaches to EV analysis to ensure sample purity and accurate attribution of biological properties. A key focus of the MISEV guidelines is the detailed reporting of exosome isolation protocols, including the techniques employed such as ultracentrifugation, chromatography, or immunoseparation, and the materials and parameters used in these processes. This level of detail is critical for ensuring reproducibility and comparability across studies. The MISEV guidelines are not specifically designed for any particular application, including skin regeneration, anti-aging, or tissue regeneration. However, the rigorous standards they establish for isolation, characterization, functional assays, and transparent reporting, serve as fundamental pillars that support the development of EV-based therapies across all biomedical disciplines, including regenerative applications.

In the context of EV research related to skin applications, however, adherence to the MISEV guidelines varies widely. While the studies reviewed highlight the considerable therapeutic potential of EVs, they also expose significant challenges, particularly in standardizing methodologies, which may hinder clinical translation. An evaluation of these studies through the framework of the MISEV guidelines reveals several areas for improvement, including the thorough reporting of isolation methods and the implementation of robust quality control measures. Addressing these gaps is essential for advancing the clinical utility of EV-based therapies.

In addition to ensuring compliance with MISEV guidelines, maintaining sterility and pyrogen-free status is a crucial factor for the safe clinical application of exosome-based products. According to GMP guidelines, all biological products intended for parenteral administration must undergo stringent sterilization and endotoxin removal processes. The purification of exosome-based formulations requires validated methods, such as sterile filtration or aseptic processing, to ensure microbiological safety. Given the increasing use of exosome-based injectables, developing standardized protocols that address sterility and regulatory compliance is critical for their successful clinical translation [73].

Ye et al. [63] demonstrated high compliance with MISEV, particularly regarding nomenclature and methodological transparency. The use of markers (CD63, CD81, TSG101) and transmission electron microscopy enabled appropriate EV characterization. Nonetheless, the lack of contamination controls (e.g., calnexin) and reliance on ultracentrifugation for EV isolation indicate areas requiring improvement under MISEV. Ersan et al. [35] utilized an aqueous two-phase system (ATPS) for isolation, which aligns with transparency principles, but the absence of contamination tests and detailed biochemical analyses reduced compliance with MISEV. Wyles et al. [41,44] exhibited limited compliance with MISEV2023, primarily due to insufficient descriptions of surface markers and isolation methods. The lack of detailed analyses on EV purity and evidence of endosomal origin restricted the interpretability of their results, despite functional studies consistent with the guidelines. Cho et al. [59] demonstrated rigorous compliance with the MISEV2023 guidelines by confirming the absence of contamination, such as undetectable levels of calnexin by ELISA, while also validating the presence of positive markers including CD63, CD9, and CD81. The isolation process utilized ultrafiltration and diafiltration through tangential flow filtration (TFF). However, the MISEV guidelines recommend incorporating additional techniques, such as density gradient ultracentrifugation, to provide a more thorough assessment of purity. Johnson et al. [42] demonstrated the highest compliance with MISEV2023 through the application of multi-step isolation (LEAP), the use of both positive (CD63, CD9) and negative markers (calnexin), and detailed functional characterization of EVs. Their approach exemplifies the standards of purity and reproducibility outlined in the guidelines. Kwon et al. [43] achieved partial compliance with MISEV2018 by utilizing appropriate nomenclature and positive markers (CD9, CD63, CD81) and excluding the presence of negative markers such as calnexin and cytochrome C. However, the single-step isolation method (ExoSCRT™) and the absence of additional EV types or contamination controls diminished their alignment with the MISEV guidelines. Svolacchia et al. [45] relied solely on ultrafiltration for EV isolation, which contradicts MISEV2023’s preference for multi-step approaches. Positive markers (CD81 and CD146) were identified, but the lack of negative marker analyses (e.g., calnexin) limits the reliability of the sample purity. Functional studies were confined to basic clinical observations, falling short of fully adhering to the MISEV2023 guidelines. 

There are also very interesting preclinical studies, such as the study by Kim et al. [31], which evaluated the effects of exosomes derived from human umbilical cord blood mesenchymal stem cells (USC-CM Exos) on skin regeneration. USC-CM Exos were characterized by their small size (average 120 nm) and high concentrations of growth factors, including EGF and basic fibroblast growth factor (bFGF). When applied topically to skin samples, the authors demonstrated that these nanostructures penetrated the stratum corneum within 3 h and reached deeper epidermal layers by 18 h. After an 8-week treatment period, increased synthesis of type I collagen and elastin was observed, along with a reduction in the expression of MMP-1. These findings highlight the ability of USC-CM Exos to restore the extracellular matrix (ECM) and rejuvenate the skin on both cellular and molecular levels. However, for such findings to be successfully translated into clinical trials, it is crucial that future studies adhere to the MISEV guidelines, ensuring their reliability and credibility in therapeutic applications.

Among the critical factors affecting the clinical success of EV-based skin therapies is the efficiency of delivery to target tissues. The utilization of EVs for skin regeneration has been tested using various delivery methods, each presenting distinct advantages and challenges. Topical formulations, such as EV-enriched creams and serums, provide a non-invasive approach. This was the most common method of application among the clinical studies analyzed in our review [41,43,44,59,60,63]. However, their effectiveness is limited by the barrier function of the stratum corneum, necessitating the use of permeation enhancers or nanocarriers to facilitate deeper penetration. Moreover, while some studies specify the exosome concentration in such formulations and treatment duration, others lack precise quantification, making comparison of their effectiveness challenging, if not impossible. We attempted to present this comparison in Table 2. If reported, exosome dosages vary significantly, ranging from 1 × 10^9^ in a study by Ersan et al. [35] to 9.78 × 10^10^ particles/mL in a study by Kwon et al. [43], with most studies utilizing twice-daily applications. Treatment windows are similarly inconsistent, spanning 4 to 26 weeks, further complicating the establishment of standardized protocols. 

In contrast, injectable administration allows for targeted delivery of EVs into the dermis or subcutaneous tissue, ensuring direct interaction with fibroblasts and hair follicle cells, which is particularly beneficial for wound healing and hair regeneration. [35,42] Techniques such as mesotherapy and microneedling have been employed to enhance EV delivery [45,62]. Among the analyzed studies, reported exosome concentrations varied considerably, ranging from 5 × 10^9^ [62] to 3 × 10^10^ EVs [35], reflecting differences in formulation and intended therapeutic effects. Johnson et al. used a different approach, administering 100 μg of pEVs, relying on protein content rather than particle number as a dosing metric. Treatment schedules also differed, from a single injection [35,42] to three sessions at 3-week intervals [62]. Svolacchia et al. [45], while lacking dosage specificity, followed a single mesotherapy session protocol.

Beyond these conventional approaches, hydrogel-based delivery systems and tissue-engineered scaffolds have been explored for sustained EV release. It seems that encapsulating EVs within biocompatible hydrogels improves their stability, moisture retention, and protection from enzymatic degradation, thereby extending the therapeutic window [36,38]. Similarly, scaffolds composed of biocompatible materials such as collagen or fibrin support cell adhesion, proliferation, and migration, prolonging EV bioactivity [29,74]. These novel delivery strategies could help maximize the therapeutic benefits of EVs, making them more viable for routine clinical use in dermatology.

Increasing research highlights the potential of exosome-based therapies in dermatology and regenerative medicine, showing benefits such as improved skin hydration, elasticity, pigmentation, and wound healing. However, the lack of standardized dosing regimens, administration routes, and treatment durations significantly hampers the development of consistent evidence-based protocols. Variations in exosome concentration, frequency of application, and treatment schedules pose substantial challenges in comparing study outcomes and translating findings into clinical practice. Moreover, the long-term effects of these therapies remain insufficiently explored, as most studies limit their assessments to a 12-week timeframe. These inconsistencies highlight the need for greater standardization in therapeutic approaches to maximize the clinical potential of exosome-based treatments.

- Regulatory and Manufacturing Challenges

Despite the growing interest and rapid progress in EV-based therapies, regulatory challenges remain a major obstacle to their widespread clinical use. Currently, no EV-based dermatological treatment has received full approval from major regulatory agencies, such as the U.S. Food and Drug Administration (FDA) or the European Medicines Agency (EMA). A major challenge is the unclear classification of EVs, which may be regulated as biologics, cell-based therapies, or drug products, each with different regulatory requirements. This uncertainty delays the approval process and slows clinical translation [71].

The absence of standardized guidelines for EV isolation, characterization, and quality control further exacerbates these regulatory challenges. While the MISEV guidelines provide a foundational framework for EV research, they fall short of addressing the stringent regulatory requirements necessary for clinical-grade EV production. Consequently, efforts are being directed toward the development of GMP-compliant production methods to ensure the consistency, safety, and efficacy of EV-based therapies [70]. However, adherence to MISEV guidelines varies considerably across studies, influencing the scientific rigor and clinical applicability of research findings. Greater compliance with these guidelines could enhance the reliability and reproducibility of clinical research, thereby advancing the integration of exosome-based therapies into dermatology and regenerative medicine.

Moreover, variations in treatment approaches, including dosing strategies, delivery methods, and safety evaluations, underscore the necessity for robust regulatory frameworks. Without standardized production protocols and clearly defined regulatory pathways, the transition of EVs from experimental treatments to approved clinical therapies remains highly challenging. Addressing these issues is imperative for the advancement of EV-based regenerative medicine.

### Limitations and Challenges

Despite promising results, the application of exosomes in aesthetic medicine and dermatology faces significant limitations. Key challenges include the need for more standardization in isolation, storage, and administration methods. Differences in research protocols hinder the comparability of results and their translation into clinical practice [75]. Another issue is the high cost of exosome therapies, which may restrict accessibility. According to GMP, producing exosomes is complex and requires advanced laboratory infrastructure, resulting in high treatment prices [76]. Additionally, clinical studies often suffer from small sample sizes, lack of diversity, and short follow-up periods, limiting the generalizability of findings. Many studies focus on narrow participant groups, such as specific genders or age ranges, and may exclude important variables like racial composition. Large, randomized clinical trials with diverse participants are needed to evaluate the long-term efficacy and safety of exosome-based therapies. Future research should focus on optimizing exosome production and storage methods to ensure their efficacy and safety [77]. Such studies are essential for establishing exosomes as a new standard in aesthetic medicine.

## 4. Methodology

The review includes clinical studies concerning the use of EVs in skin conditions published in English up to November 2024. Studies evaluating the efficacy of EVs in anti-aging therapies, acne scar treatment, alopecia, or wound healing were considered. Only studies conducted on humans or using preclinical models with high clinical translational potential were included. Studies that focused exclusively on in vitro experiments without human in vivo models or presenting low levels of evidence, such as case reports or editorial comments, were excluded.

The literature was searched across three main scientific databases: PubMed, Scopus, and Web of Science. The search was conducted using combinations of keywords such as “extracellular vesicles”, “exosomes”, “aesthetic medicine”, “skin rejuvenation”, “alopecia”, “acne scars”, and “wound healing”, with appropriate logical operators (AND, OR). Additionally, the bibliographies of selected articles were analyzed to identify potentially overlooked studies. The obtained results were initially screened based on titles and abstracts. Three independent reviewers conducted a full-text review of the selected studies, applying predefined inclusion and exclusion criteria.

Study findings were grouped by application areas: anti-aging therapies, acne scar regeneration, alopecia treatment, and wound healing. Each of these areas underwent a detailed analysis, considering clinical and preclinical study outcomes and identifying research gaps. The details and results of the analyzed studies are summarized in Table 2. It summarizes the results of 11 clinical studies conducted on human participants, detailing the leading institution, start date, origin, dosage, and administration method of EVs.

## 5. Conclusions

Exosomes are a promising tool in aesthetic medicine and dermatology, offering wide therapeutic possibilities in skin regeneration, alopecia treatment, acne scar healing, and wound care. Available research indicates that exosomes can modulate key biological processes such as angiogenesis, fibroblast proliferation, and collagen synthesis, making them an attractive alternative to more invasive methods. However, further research is necessary to establish exosomes as a therapeutic standard. Large, randomized clinical trials are essential to assess their long-term efficacy and safety. Developing standards for exosome isolation, storage, and application will also enable better comparability of study results and enhance clinical translation.

Despite their promise in promoting skin health and regenerative processes, several challenges hinder the clinical translation of exosome-based therapies. Developing standardized dosing protocols, establishing comprehensive guidelines for EV isolation and characterization, and clarifying regulatory classifications are essential steps for advancing this field. Addressing these challenges is crucial for the full realization of the therapeutic potential of exosome-based interventions.

## Figures and Tables

**Table 1:** Product of research.

Product of Research	Correct Nomenclature	Morphology		Characterization		Isolation Method	Author	Year
		Size [nm]	Analysis Method	Positive Markers	Negative Markers			
Human foreskin-derived mesenchymal stromal cell derived exosomes	−	139.7 ± 2.3	NTA	CD9, CD63, CD81	NA	ATPS	Ersan et al. [35]	2023
Human umbilical cord mesenchymal stem cell-derived exosomes	+	40–80	TEM, NTA	CD63, CD81, TSG101	NA	Ultrafiltration	Ye et al. [63]	2022
Allogeneic platelet-derived extracellular vesicles	+	65–400	Cryo-TEM	CD63, CD9	Calnexin	LEAP	Johnson et al. [42]	2023
Human adipose tissue stem cell-derived exosomes	+/−	mean 117.4	NTA, Cryo-TEM	CD9, CD63, CD81	Calnexin, cytochrome C	ExoSCRT™	Kwon et al. [43]	2020
Human adipose tissue stem cell-derived exosome-containing solution	+/−	mean 90	Filtration analysis, flow cytometry, NTA	CD81, CD146	NA	Ultrafiltration, MACS technology	Svolacchia et al. [45]	2024
Human adipose tissue-derived stem/stromal cells	+	30–200; mean 138	NTA, membrane filtration	CD9, CD63, CD81	Calnexin	Ultrafiltration, TFF	Cho et al. [59]	2020

(−)—non-compliant; (+)—compliant; NTA—Nanoparticle Tracking Analysis; NA—not available; ATPS—Aqueous Two-Phase System; TEM—Transmission Electron Microscopy; LEAP—Ligand-based Exosome Affinity Purification; MACS—Magnetic-Activated Cell Sorting; TFF—Tangential Flow Filtration;.

**Table 2:** Summary of the reviewed clinical trials.

Purpose of Regenerative Therapy	Study Status	Start Date	Institution	Origin of Exosomes	EV/ Exosome Dosage	Route and Administration	Treatment Window	Follow-Up Period	Number of Patients	Age Range [Years]	Results	Compared to	Authors, Year
Skin Brightening	Concluded	NDA	ExoCoBio Exosome Institute	ASCs	0.2 g of test with 2.0 × 10^10^ ASC-exosomes/mL applied twice daily	Topical application	8 weeks	8 weeks	21	39–55	Statistically significant decrease in melanin content on the exosome-treated side compared to placebo, with the effect diminishing over time	Placebo	Cho et al., 2020 [59]
Skin Rejuvenation	Concluded	October 2021	Mayo Clinic	HPE	NDA, applied twice daily	Topical application	6 weeks	6 weeks	56	40–80	Significant improvements in skin health score, reduction in redness, wrinkles, and melanin production; enhanced luminosity and color evenness	NA	Proffer et al., 2022 [60]
Hands Photoaging	Concluded	NDA	Mayo Clinic	HPE	NDA, applied twice daily	Topical application, twice daily	12–26 weeks	26 weeks	60	40–80	Improvements in skin texture and reduced wrinkles with both treatments; similar results in reducing brown spots and wrinkles between HPE and vitamin C	Vitamin C treatment	Wyles et al., 2024 [44]
Skin Rejuvenation	Concluded	1 October 2021	Mayo Clinic	HPE	NDA, applied twice daily	Topical application	12 weeks	12 weeks	56	40–80	Improvements in skin aging, pigment reduction, luminosity, and color evenness reported by the majority of participants; no serious adverse effects reported	Skin biopsies of the same subjects at baseline	Wyles et al., 2024 [41]
Skin Rejuvenation	Concluded	NDA	DeNova Research	HPE	NDA, applied three times daily	Topical application	4 weeks	4 weeks	18	32–77	Brighter and more youthful-looking skin reported by participants treated with the serum compared to the control group, with less crusting post procedure	Control group treated with post-procedural standard of care	Dayan et al., 2023 [61]
Tissue Regeneration and Antiaging	Concluded	NDA	University of Rome	ADSCs	NDA	Intradermal injection	Single treatment session	6 months	72	34–68	Enhanced tissue regeneration, improved skin texture, and reduction in wrinkles; no adverse effects reported	NA	Svolacchia et al., 2024 [45]
Facial Skin Aging	Concluded	NDA	Chung-Ang University College of Medicine	ASCs	5 × 10^9^ ASCE particles	Topical application combined with microneedling	Three treatment sessions at 3-week intervals	12 weeks	28	43–66	Significant improvement in skin wrinkles, elasticity, and hydration on the exosome-treated side	Microneedling alone	Park and Kwon, et al., 2023 [62]
Sensitive Skin Treatment	Concluded	NDA	The Third Affiliated Hospital of Sun Yat-sen University	USC-CMs	1 mL exosome product, concentration unspecified, applied twice daily	Topical application	4 weeks	4 weeks	22	24–55	Improved skin hydration, reduced redness, and enhanced skin barrier function; no adverse effects reported	NA	Ye et al., 2022 [63]
Acne Scar Treatment	Concluded	NDA	Oaro Dermatology Institute	ASCs	9.78 × 10^10^ particles/mL on treatment day, 1.63 × 10^10^ particles/mL twice daily for two days post-FCL	Topical application post- FCL	Three treatment sessions at 3-week intervals	12 weeks	25	19–54	Greater improvement in acne scars on exosome-treated side; milder erythema and shorter downtime	Control gel	Kwon et al., 2020 [43]
Androgenetic Alopecia	Concluded	1 January 2024	Yeditepe University Hospital	Foreskin-derived MSCs	1 × 10^10^ EVs per mL, total of 3 mL suspension	Intradermal scalp injection	Single treatment session	12 weeks	30	22–65	Statistically significant increase in hair density; high patient satisfaction; no side effects observed	NA	Ersan et al., 2024 [35]
Delayed Wound Healing	Concluded	22 September 2020	Exopharm Ltd.	pEVs	100 μg LEAP-isolated pEVs in 340 μL isotonic buffer	Subcutaneous injection	Single treatment session	30 days	11	NDA, mean age: 29	Quick healing of all wounds, without time differences compared to placebo; injections deemed safe and well tolerated	Placebo	Johnson et al., 2023 [42]

(NDA—No Data Available; ASCs—Adipose Tissue Stem/Stromal cells; HPE—Human Platelet Extract; NA—Not Applicable; ADSCs—Adult Stem Cells; ASCE—Adipose tissue Stem Cell-derived Exosomes; USC-CMs—Umbilical Cord Mesenchymal Stem Cells; FCL—Fractional Carbon Dioxide Laser treatment; MSCs—Mesenchymal Stem Cells; EVs—Extracellular Vesicles; pEVs—platelet-derived Extracellular Vesicles; LEAP—Ligand-based Exosome Affinity Purification).
